# DNA identification of compromised samples with massive parallel sequencing

**DOI:** 10.1080/20961790.2018.1509186

**Published:** 2018-10-29

**Authors:** Andreas Tillmar, Ida Grandell, Kerstin Montelius

**Affiliations:** a Department of Forensic Genetics and Forensic Toxicology, National Board of Forensic Medicine, Linköping, Sweden;; b Department of Clinical and Experimental Medicine, Faculty of Health Sciences, Linköping University, Linköping, Sweden

**Keywords:** Forensic science, forensic genetics, disaster victim identification, massive parallel sequencing, SNP, mass disaster

## Abstract

Genetic profiling is a standard procedure for human identification, i.e. in criminal cases and mass disasters, and has been proven to be an important part in the process in the repatriation of victims to their relatives. In the event of a catastrophe whether it be a natural disaster, terror attack or accident, fatalities of many nationalities may be a consequence and international collaboration becomes necessary. Current DNA techniques used on a routine basis at forensic laboratories world-wide are very useful, and results reported from different labs are compared, making it possible to be matched in order to declare the identification of a victim. Statistical calculations of possibilities of a random match are achievable since population data from many parts of the world are available. However, decomposition and degradation of the remains are not uncommon in the aftermath of a catastrophe and hence it may be difficult to retrieve detailed DNA profiles from such samples. Massive parallel sequencing (MPS) is a technique capable of producing a vast amount of DNA sequence data in a high-through put manner, and panels of single nucleotide polymorphism (SNP) markers allow the amplification of small DNA fragments, often seen in compromised samples. Here, we report the results from a set of 10 samples from missing person identification cases, analyzed with an MPS based method comprising 131 SNP markers and compared with direct reference material or buccal swab samples collected from relatives of the deceased. We assess the weight of evidence of a match by statistical calculation. Furthermore, we compare results reported on different platforms using different SNP panels, and conclude that more work has to be done if results from missing person identification cases analyzed on MPS with SNP panels at different laboratories are to be fully reliable and thus comparable.

## Introduction

DNA profiling is one of the primary methods by which victims of a disaster can be identified, together with odontology and ridgeology [1]. Capillary electrophoresis (CE) of short tandem repeats (STRs) has been the choice of methodology in DNA identification for more than 20 years [2–4]. In daily routine work, CE and STRs have the capability to solve most cases with the required power of exclusion, with the markers available on the market. However, the CE technique has its limitations. There is an issue in multiplex capability, i.e. how many markers it is possible to analyze simultaneously, given the power of exclusion needed in deficiency cases or in a disaster with many victims. Also, the nature of STR polymorphisms [5] means that relatively long amplicons are needed to even obtain any result at all. In a disaster, the circumstances of the incident may cause vast damage to the surroundings as well as to the samples to be examined. DNA extracted from such samples may be degraded and only yield short fragments. Standard techniques may not be sufficient to analyze such compromised samples.

Single nucleotide polymorphisms (SNPs) are markers found in abundance within the human genome [6]. Many have been designated to certain applications, such as predictions of phenotypical traits, identification and kinship analysis [7–12]. The amplicons needed to analyze the SNPs are shorter (less than 100 base pairs) than those needed for STRs. They are also less prone to mutations than STRs but hold less information due to their lower genetic diversity [13]. Therefore, in order to obtain similar informative values as for STRs, a larger number of SNPs are required for human identity testing and several multiplex assays have been described [9,14–16].

Massive parallel sequencing (MPS) has proven to be a high-throughput technique which maintains high-quality standards of reproducibility and produces reliable results. However, 100% accuracy cannot be guaranteed [17]. The capacity of MPS technology permits the simultaneous analysis of a much larger number of markers compared with CE-based methods [8]. This fact increases the capability to extract data from samples with damaged and minute amounts of DNA. Identification of a victim follows the matching of a postmortem (PM) sample to an antemortem (AM) sample and the samples need to be analyzed according to the same parameters to be comparable. In many countries, buccal swabs or blood on Flinders Technology Associates (FTA) cards are standardized samples used on a daily basis. In order to keep the workflow as efficient as possible, it is important to allow FTA card samples to be used as the starting material for MPS based assays. This has been demonstrated earlier to be possible both with DNA extracted from the cards and with direct amplification using standard CE based analysis methods [18]. In order to assess the weight of evidence, it is also necessary to possess relevant allele frequency databases of the population to allow the statistical evaluations. Population databases of the different SNP-multiplexes are available [6,19,20].

Here, we describe compromised bone and tissue samples from missing person identification cases which are analyzed with 131 multiplexed SNPs on an MPS platform and the outcome is compared with the analysis of 15 STRs analyzed with CE. The aim was to investigate whether the MPS assay, in its presented form [21], could be used to obtain reliable SNP profiles for samples normally found in disaster victim identification (DVI) or missing person identification cases.

## Material and methods

The custom made SNP panel originally consisted of 140 SNPs of which 52 were chosen from the SNP*for*ID project [22] and the remaining 88 SNPs were chosen from the study by Pakstis et al. [23]. A more detailed description of the marker panel can be found in Grandell et al. [21]. Nine of the 140 SNP markers were excluded after the validation of the assay; thus, 131 SNPs were included in the analyses below.

Ten samples were chosen for the analysis of which eight were femur and two were soft tissues (Table 1). The DNA from the femur samples was extracted using a phenol/chloroform based extraction method [24]. The DNA from the soft tissues were extracted with QIAamp DNA blood Mini Kit (Qiagen, Hilden, Germany). DNA concentration was measured with NanoDrop spectrophotometer (Thermo Fisher Scientific, Waltham, MA, USA) and ranged from not detectable levels to 100 ng/µL (Table 1).

**Table 1.** Summary and comments of the result from the analysis of remains from 10 missing persons.

Case No.	Sample type (PM)	DNA concentration (ng/µL)	Reference (AM)	Result from STR analysis with CE*	LR based on the STR data	Comments on STR profile quality	Result from SNP analysis with MPS*	LR based on the SNP data	Comments on SNP profile quality
1	Femur	–	Full sibling	10/15 STRs	1.2 × 10^4^	Partial DNA profile, 5 genotypes excluded due to peak height imbalance or low peak heights	122/131 SNPs	2.5 × 10^7^	Partial DNA profile, 8 genotypes excluded due to low coverage and/or imbalanced allele calls
2	Tumor tissue	41	Same individual	0/15 STRs	–	No DNA profile. Low peak heights and/or >2 peaks (drop-in) per marker	96/131 SNPs	3.1 × 10^40^	Partial DNA profile, 35 genotypes excluded due to low coverage and/or imbalanced allele calls
3	Fat tissue	22	Same individual	0/15 STRs	–	No DNA profile. Low peak heights and/or >2 peaks (drop-in) per marker	73/131 SNPs	3.8 × 10^30^	Partial DNA profile, 58 genotypes excluded due to low coverage and/or imbalanced allele calls
4	Femur	12	Monozygotic twin	15/15 STRs	1.4 × 10^26^	Full DNA profile	126/131 SNPs	1.7 × 10^54^	Partial DNA profile, 5 genotypes excluded due to low coverage and/or imbalanced allele calls
5	Femur	–	Full sibling	0/15 STRs	–	Sporadic peaks, not reproducable	0/131 SNPs	–	All genotypes excluded due to low coverage and/or imbalanced allele calls
6	Femur	–	Same individual	0/15 STRs	–	Sporadic peaks, not reproducible	0/131 SNPs	–	All genotypes excluded due to low coverage and/or imbalanced allele calls
7	Femur	6	No reference material	0/15 STRs	–	Sporadic peaks, not reproducible	67/131 SNPs	–	Partial DNA profile, 8 genotypes excluded due to low coverage and/or imbalanced allele calls
8	Femur	100	Parent/child	15/15 STRs	7.0 × 10^5^	Full DNA profile	130/131 SNPs	4.3 × 10^10^	Partial DNA profile, 1 genotype excluded due to low coverage and/or imbalanced allele calls
9	Femur	20	Parent/child	15/15 STRs	2.3 × 10^5^	Full DNA profile	131/131 SNPs	1.5 × 10^12^	Full DNA profile
10	Femur	3	Half-uncle	15/15 STRs	12	Full DNA profile	131/131 SNPs	40	Full DNA profile

PM: postmortem; AM: antemortem; STR: short tandem repeat; CE: capillary electrophoresis; LR: likelihood ratio; MPS: massive parallel sequencing; SNP: single nucleotide polymorphism; –: not detectable; *Number of markers that met the quality criteria/Number of markers included in the analysis.

The MPS analysis was performed as described in Grandell et al. [21]. In brief, the DNA libraries were constructed using the GeneRead^TM^ DNAseq Targeted Panels V2 library preparation workflow (Qiagen) from an initial PCR amplification using a custom made primer mix. The initial amplification was performed using 24 PCR cycles. Totally, 8 µL of extracted DNA were used as template. If the DNA concentration was above 2.5 ng/µL, the sample was diluted to 2.5 ng/µL from which 8 µL was taken. If the DNA concentration was below 2.5 ng/µL, 8 µL was taken without any dilution. The libraries were prepared following the manufacturer’s protocol with 12 samples multiplexing including one positive control (2800M) and one negative control (nuclease free H_2_O). Quality measurements were performed twice during the library preparation using the 2100 Bioanalyzer (Agilent, Santa Clara, CA, USA). Firstly after the purification of the initial PCR amplification, and secondly after the final purification following the library amplification. The quality measurements were used to control that all primer-dimers and adapter-dimers were purified, and to confirm the expected size distribution of the PCR product and the library, respectively. The final libraries were quantified using Qubit 2.0 Fluorometer (Thermo Fisher Scientific), diluted, normalized and pooled together to a final concentration of 4 nmol/L. The sequencing was performed on a MiSeq (Illumina, San Diego, CA, USA) with Reagent Kit v3.

After the sequencing, sample demultiplexing was performed on the MiSeq Reporter (Illumina) and the output (.fastq-files) was used as the input for the bioinformatic analyses using the Biomedical Genomics Workbench v 2.1.1 (CLC Bio, Qiagen). The SNP detection was performed with the “Known mutation from a sample”-workflow included in the software with hg19 as the reference genome. The minimum coverage for genotype calling was set to 200× and heterozygote balance was analyzed based on allele read frequency (ARF) values. Allele read frequency values between 0 and 0.1 and between 0.9 and 1 were used as thresholds for inclusion of a homozygous genotype and ARF between 0.4 and 0.6 was used as threshold for inclusion of a heterozygous genotype. The final allele call was performed in an in-house made Excel spreadsheet.

For comparison, the DNA samples were also analyzed with STR markers using PCR and CE. The AmpFℓSTR Identifiler PCR Amplification Kit (Life Technologies, Carlsbad, CA, USA) was used for amplification and the PCR products were then analyzed on an AB3500xL (Life Technologies) according to the manufacturers protocol. Totally 1 µL of 1 ng/µL concentrated DNA was used as input for the STR analysis. If the DNA concentration was less than 1 ng/µL, a maximum of 2 µL of the DNA elute was used as input.

### Statistical calculations

For each one of the cases, the likelihood ratio (LR) was calculated from the obtained SNP and STR profile data given the case hypothesis. The software Familias [25] was used for LR calculation based on Swedish allele frequencies [21,26]. The software FamLink was used in order to account for genetic linkage between closely located loci [21,27]. Linkage was considered when the distance between adjacent markers (STR-STR, STR-SNP, SNP-SNP) was shorter than 50 centimorgans.

## Results and discussion

The results in this report describe the outcome of 10 missing person identification cases analyzed with both CE and MPS marker panels, and their comparison. Both the PM and AM samples have been analyzed using the same techniques. Four samples resulted in complete STR profiles using CE (Table 1). Five of the samples did not yield any STR profiles using CE. This was either due to absence of detectable peaks in the electropherograms, peaks below quality thresholds, drop-in peaks and/or samples with sporadic peaks that were not reproducible (Table 1). One sample resulted in a partial STR profile, for which some genotypes did not meet the quality thresholds for peak heights and/or peak height balance. No clear correlation was observed between the DNA concentration of the samples and the results of the STR profile analysis.

Two of the samples resulted in full SNP profiles, and an additional four samples showed SNP profiles where only 1–8 genotypes per sample failed to meet the quality criteria (Table 1). Two of the samples did not give any SNP profiles at all, and for two samples, partial SNP profiles were obtained with approximately 26.7% and 44.3% missing genotypes respectively (Table 1).

**Figure 1.**
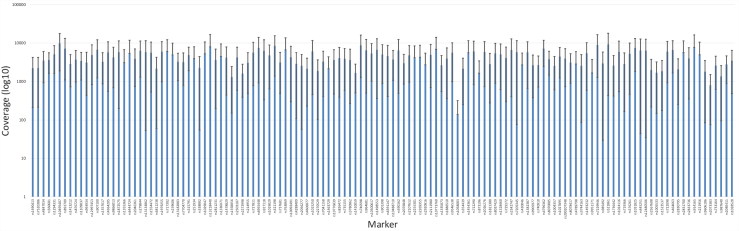
Mean and standard deviation of the coverage for each of the single nucleotide polymorphism (SNP) marker included in the analysis using massive parallel sequencing (MPS). The chart is based on the eight postmortem (PM) samples that resulted in full or partial SNP profiles.

**Figure 2.**
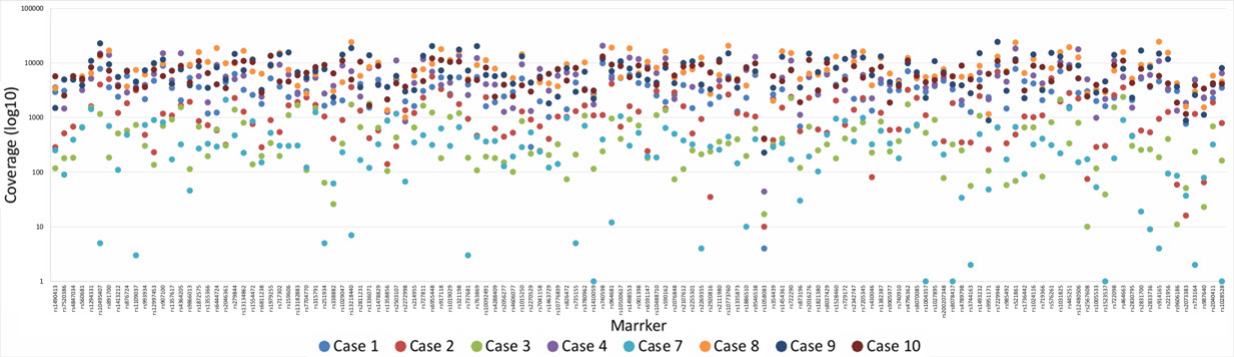
Sample and marker specific coverage for the eight postmortem (PM) samples that resulted in full or partial single nucleotide polymorphism (SNP) profiles for the massive parallel sequencing (MPS) analysis. A coverage of 200× was used as a minimum threshold for genotype calling.

**Figure 3.**
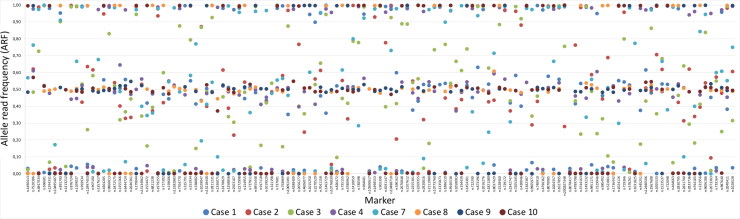
Sample and marker specific allele read frequency (ARF) for the eight postmortem (PM) samples that resulted in full or partial single nucleotide polymorphism (SNP) profiles for the massive parallel sequencing (MPS) analysis. An ARF values between 0.4 and 0.6 resulted in a heterozygous genotype and an ARF value between 0 and 0.1 or between 0.9 and 1 resulted in a homozygous genotype.

The two quality parameters, coverage and ARF (e.g. allele balance), were used for genotype calling for the SNP based MPS analysis, as noted in the material and method section. In general, the coverage was high for all markers except for the SNP marker rs1058083 (Figure 1). However, some sample specific variations of the coverage were observed (Figure 2). Similar patterns were also observed for the allele balance calling (Figure 3). As expected, the samples that failed to produce any SNP data or showed partial SNP profiles were unbalanced. Supplementary Figures 1 and 2 show examples of outputs for PM samples that resulted in a good-quality SNP profile or a bad-quality SNP profile. Different quality measurements need to be considered when evaluating data from MPS analysis compared with interpreting DNA profiles from CE analysis.

When comparing the data between the CE and the MPS analysis of the 10 samples described, it was shown that SNP MPS was successful in three of the cases where STR CE failed. The SNP analysis on MPS was based on a larger amount of DNA than the STR CE analysis. However, we do not believe that this was the reason for the improved results with the SNP analysis. A more probable reason was the increased sensitivity with MPS based methods, and/or the advantage of SNP markers (biallelic markers and shorter amplicons) over STR markers (multiallelic and longer amplicons) for possibly degraded samples. As noted above, the aim herein was to study the current MPS based method's ability to obtain reliable SNP data of degraded samples. Further optimizations are possible in order to improve the method for degraded and low-quantity DNA samples such as the PM samples in missing person casework. Such optimizations could involve altering PCR cycling numbers, improving purification steps as well as changing the primer design of the panel.

The obtained SNP and STR profiles were, together with the case hypothesis, used to calculate case specific LRs. In all cases but one, where full or partial SNP and STR profiles were available, high LRs were obtained (Table 1). In one case, comprising a half-uncle/half nephew relationship, only a limited statistical support was obtained.

In the event of a major incident, it is not unlikely that the identification process will include many nationalities and reference samples collected from many countries. A number of evaluations have been reported on MiSeq (Illumina), Ion Torrent and HID-Ion PGM (Thermo Fisher Scientific) [21,28–31]. These studies report results on different platforms, different sample types and marker sets. One study reports the comparison between MiSeq FGx^TM^ and HID-Ion PGM^TM^ using multiplexes of 173 and 124 SNPs respectively. Eighty-three of the SNPs were common for both platforms and genotype calls showed 99.7% concordance between the platforms [32]. However, three of the common SNPs showed a non-concordance of more than 4.8%. Also, the two platforms use different nomenclature reporting the allele calls. As for the STR markers, the SNPs included in marker panels have to be coordinated between countries and laboratories, and different MPS platforms and bioinformatic pipelines must generate comparable results. These data show the necessity to discuss both SNP multiplexes and MPS platforms on international terms.

## Conclusion

As shown in this study, the MPS technique and SNP panels may be used to solve complicated cases. The technique has the capability to sequence compromised samples where standard STR CE methods fail. This knowledge gives the opportunity to solve identification cases that earlier have been considered difficult. Reported investigations show that a variety of sample types are tested and verified on some of the instruments available. In addition, different laboratories use different SNP panels, which means there are several combinations of instruments, sample types and SNP panels running in different labs. In the event of an international identification operation, involved laboratories need to analyze the samples in question in a comparable way. The community needs more knowledge about the concordance between the different MPS sequencing methodologies in order to be able to share DNA profiles between laboratories for identification purposes.
